# Complete mitogenomes for two lineages of the Australian smelt, *Retropinna semoni* (Osmeriformes: Retropinnidae)

**DOI:** 10.1080/23802359.2016.1209097

**Published:** 2016-09-05

**Authors:** Daniel J. Schmidt, Md. Rakeb-Ul Islam, Jane M. Hughes

**Affiliations:** Australian Rivers Institute, Griffith University, Nathan, QLD, Australia

**Keywords:** Freshwater fish, mitochondrial genome, MiSeq, cryptic species, next-generation sequencing

## Abstract

Complete mitochondrial genome sequences were determined for two lineages (“CEQ” and “SEQ”) of the Australian smelt, *Retropinna semoni*. Both mitogenomes contain the typical vertebrate arrangement of 13 protein-coding genes, 22 tRNA genes, 2 rRNA genes and control region. A conventional start codon for ND2 was not present in either lineage; instead CTG (Leucine) was present at this position. These sequences will be a useful resource for evolutionary studies of a significant species complex in the Australian freshwater fish fauna.

The Australian smelt (*Retropinna semoni*) is an Australian endemic freshwater fish, widely distributed across the southeast of the continent. Genetic analyses using allozymes, microsatellites and mtDNA indicate this species may comprise a complex of at least five taxa (Hammer et al. 2007; Hughes et al. 2014). Here, we present complete mitochondrial genome sequences of two northern lineages that correspond geographically to informal “CEQ” and “SEQ” groups designated by Hammer et al. (2007). Genomic DNA was isolated from tissue voucher GUB433 (= lineage SEQ, Twin Bridges Reserve, Brisbane River, −27.430457 152.639357) and voucher GUM433 (= lineage CEQ, Conondale Bridge, Mary River, −26.727511 152.713604). DNA was sheared to an approximate mean length of 400 bp and an Illumina MiSeq-compatible sequencing library was prepared using the iTru protocol (Travis Glenn, pers. comm.). Sequencing was performed on a MiSeq instrument (Illumina, San Diego, CA), producing 2 × 300 bp paired-end reads. The two libraries each generated 1.18 × 10^7^ paired-end reads. Mitogenome assembly was performed using Geneious v9.1.5 (Kearse et al. 2012). For each taxon, overlapping paired reads were merged using the BBMerge tool and ∼6 × 10^5^ merged reads in size range 250–590 bp were sampled. Reference-guided assembly was implemented in Geneious v9.1.5 (Auckland, New Zealand) using the iterative map to reference function with *Retropinna retropinna* as reference sequence (GenBank accession: AP004108; Ishiguro et al. 2003). *De Novo* assembly was implemented in Geneious using medium sensitivity settings and allowing contigs with matching ends to circularize. A putative mitogenome was identified as the longest contig with circular topology. The putative mitogenome assembly was visually checked for errors derived from heteroplasmy or paralogues using the Geneious genome browser. An initial annotation was achieved using MitoFish (Iwasaki et al. 2013) and subsequently inspected by eye.

The complete mitogenome of *Retropinna semoni* “lineage CEQ” (GenBank accession: KX421785) was 16,582 bp in length, based on 5755 reads and mean coverage of 105. The complete mitogenome of *R. semoni* “lineage SEQ” (GenBank accession: KX421784) was 16,577 bp in length based on 13,412 reads and mean coverage of 234. Both mitogenomes contained 13 protein-coding genes, 22 tRNAs, 2 rRNAs and a control region in the standard vertebrate order. A start codon for ND2 was not determined for either of the new mitogenomes. The first ND2 codon was annotated as CTG (Leucine) in both taxa despite the presence of a conventional ATG start codon in the reference sequence. Mean read depth at the first codon position of ND2 was >97, with >99% pairwise identity. Pairwise divergence between CEQ and SEQ lineages across the full mitogenome was 3.6%, ranging from 1.8% for ATPase 6, up to 7.3% for ND6. Phylogenetic analysis using complete mitochondrial genome matches from GenBank showed the closest relationship of the two new mitogenomes with the congeneric New Zealand smelt, *Retropinna retropinna* (Figure 1). The new *R. semoni* mitogenomes (GenBank: KX421785; KX421784) matched with 100% identity to fragments of cyt b from *R. semoni* collected in the Brisbane and Mary River catchments (GenBank: JX914063; JX914057; see Page & Hughes 2014).

**Figure 1.**
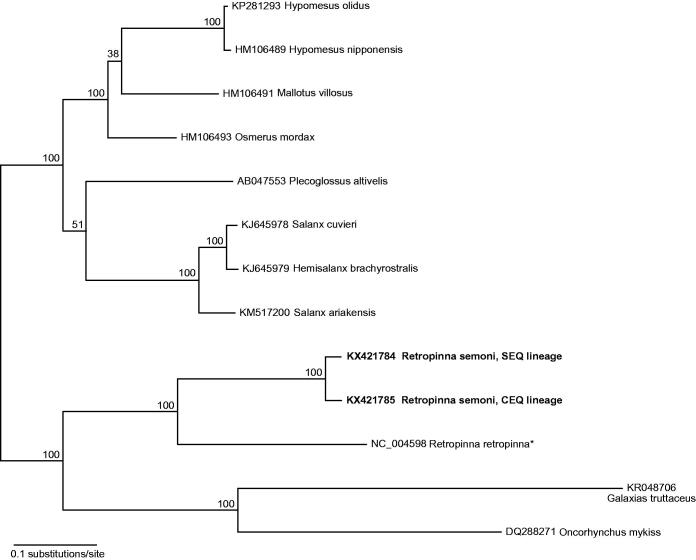
Phylogenetic placement of two *Retropinna semoni* mitogenomes with the top mitogenome hits from a blastn query of the NCBI nucleotide database. Tip labels include GenBank accession number with species name and node labels show bootstrap results. New mitogenome sequences highlighted in bold font; reference genome used for assembly denoted by an asterisk. Alignment of mitogenomes was performed using MAFFT v7.017 (Katoh et al. 2002) and excluded the d-loop which aligned poorly across taxa. A maximum likelihood phylogenetic analysis was performed on the final alignment of 15,805 bp with RAxML v7.2.8 using the GTR + GAMMA substitution model with 1000 bootstrap replicates (Stamatakis 2006).
